# Healthy Lifestyle Score and Glycemic Control in Type 2 Diabetes Mellitus Patients: A City-Wide Survey in China

**DOI:** 10.3390/healthcare11142037

**Published:** 2023-07-16

**Authors:** Mengmeng Che, Qin Zhou, Weiquan Lin, Yunou Yang, Minying Sun, Xiangyi Liu, Hui Liu, Caixia Zhang

**Affiliations:** 1 Department of Epidemiology, School of Public Health, Sun Yat-sen University, Guangzhou 510080, China; chemm@mail2.sysu.edu.cn; 2 Department of Basic Public Health, Guangzhou Center for Disease Control and Prevention, Guangzhou 510440, China; gzcdc_zhouq@gz.gov.cn (Q.Z.); gzcdc_linwq@gz.gov.cn (W.L.); gzcdc_yangyo@gz.gov.cn (Y.Y.); gzcdc_sunmy@gz.gov.cn (M.S.); gzcdc_liuxy@gz.gov.cn (X.L.)

**Keywords:** type 2 diabetes, healthy lifestyles, glycemic control, interactive effect

## Abstract

Background: Few studies have investigated the combined impact of healthy lifestyle factors on glycemic control. Our study aimed to examine the associations of a healthy lifestyle score (HLS) with glycemic control and to explore the interactive effects of lifestyle factors among patients with type 2 diabetes mellitus (T2DM) in China. Methods: This cross-sectional study was conducted among T2DM patients based on the health management of residents from Guangzhou, China. Good glycemic control was defined as fasting plasma glucose < 7.0 mmol/L. HbA1c < 7.0% was also defined as good glycemic control in sensitivity analysis. The HLS was defined as including physical activity, waist circumference, body mass index, dietary habit, smoking, and alcohol consumption. Logistic regression models were used to examine the associations and interactions between the lifestyle factors and glycemic control. Results: Compared with participants with an HLS ≤ 2, the odds ratios (95% confidence intervals) for an HLS of 3, 4, 5, and 6 were 0.82 (0.77–0.87), 0.74 (0.70–0.79), 0.61 (0.57–0.65), and 0.56 (0.53–0.60), respectively. Significant interactions of healthy lifestyle factors in relation to glycemic control were shown (*P_interaction_* < 0.05). Conclusions: A healthier lifestyle was significantly associated with good glycemic control in patients with T2DM, and combined healthy lifestyle factors had a better effect than considering them individually.

## 1. Introduction

In 2021, 537 million people were diagnosed with diabetes, and more than 90% of them had type 2 diabetes mellitus (T2DM) [1]. All-cause mortality is almost twice as high as for people without diabetes among patients younger than 55 years and with a glycated hemoglobin Alc (HbA1c) level of 6.9% or less, and this risk increases to more than four times with rising HbA1c [2].

Accumulating evidence has revealed the importance of glycemic control for T2DM patients. Lower blood glucose is related to a lower risk of death among patients with T2DM [3,4]. In addition, good glycemic control might enable T2DM patients to prevent or delay complications related to diabetes, and to improve their quality of life [4,5]. Treatments for diabetes include maintaining a healthy lifestyle and taking anti-hyperglycemic drugs. Because of the side effects and high cost of long-term medications [6], a healthy lifestyle is essential for patients with diabetes to manage their blood glucose levels. However, previous studies failed to observe a significant association between lifestyle factors and blood glucose levels [7,8]. Owing to the complexity of blood glucose metabolism, the effect of single healthy lifestyle factors on blood glucose control is limited. Therefore, a combined healthy lifestyle may be of greater benefit to patients with T2DM.

Engaging in a combined healthy lifestyle has many health benefits, including preventing chronic non-communicable diseases [9,10] and lowering mortality [11,12]. Some studies have indicated that lifestyle factors are typically correlated with one another. For example, smoking is related to other unhealthy lifestyles [13,14]. Moreover, interactive effects may exist between lifestyle factors [15]. Therefore, we suppose that a combined healthy lifestyle may have a stronger effect on glycemic control than considering the elements individually.

To the best of our knowledge, few studies have investigated the combined impact of healthy lifestyle factors on glycemic control. A systematic review in 2015 [16] analyzed 11 randomized controlled trial studies and reported that lifestyle weight-loss intervention including an energy-restricted diet and regular physical activity had nonsignificant beneficial effects on HbA1c. However, most studies mainly focused on energy restriction and only two studies simultaneously included increased level of physical activity. A randomized controlled trial in 2017 [17] failed to observe a significant reduction in HbA1c using intensive lifestyle intervention (increased physical activity and decreased diet intake to lose weight) in overweight or obese patients with T2DM, compared with those who received standard healthcare. A prospective cohort study in Germany [18] found a nonsignificant relationship between glycemic control and the total score of lifestyle behaviors, including those relating to no smoking, no alcohol consumption, adequate physical activity and a healthy diet. A cross-sectional study in China [19] also failed to find significant differences in glycemic control between participants with the lowest lifestyle score and higher lifestyle score, including adequate physical activity, normal waist circumference, normal body mass index (BMI), healthy dietary habits, no smoking and no alcohol consumption. In the China Kadoorie Biobank study, only 25.2% of the participants adopted the healthiest lifestyles [20]. Therefore, great importance should be attached to the promotion of the combined healthy lifestyle.

In this context, this study aimed to use a city-wide survey to examine combined lifestyle factors in relation to glycemic control among patients with T2DM in China. We also explored the interactive effects between one of the healthy lifestyle factors and the combination of the other factors for the risk of poor glycemic control.

## 2. Materials and Methods

### 2.1. Data Source and Study Population

**Figure 1:**
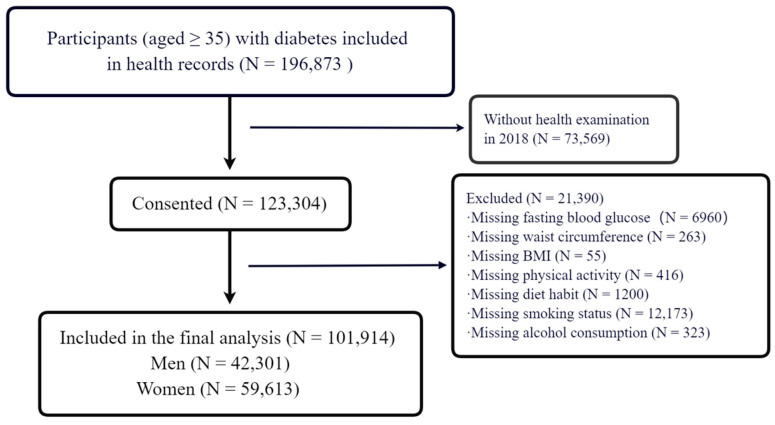
Flow chart of participant selection.

The raw database of this study was collected based on the health management of residents with T2DM, one of the National Basic Public Health Services, in Guangzhou, China. The inclusion criteria for health management of residents with T2DM service were as follows: residents diagnosed with T2DM; people who had lived in Guangzhou for more than 6 months; and people aged 35 or above. For those residents who received health management for their T2DM, free physical examinations were provided once a year. In 2018, a total of 196,873 patients with T2DM in Guangzhou received health management. For this study, 73,569 participants were excluded because they had failed to complete the health examinations, and 6960 participants were excluded because of missing fasting plasma glucose (FPG) readings. Other reasons for exclusion included missing information regarding waist circumference, BMI, physical activity, dietary habit, smoking status, and alcohol consumption (Figure 1). The final analysis included 101,914 participants.

This study was performed in accordance with the ethical standards of the Declaration of Helsinki, and all procedures were approved by the Ethical Committee of the School of Public Health, Sun Yat-sen University (approval number 2023-007).

### 2.2. Data Collection

Information on lifestyle factors (physical activities, smoking status, alcohol consumption, and dietary habits) and socio-demographic characteristics was collected by trained medical staff with questionnaires through face-to-face interviews. Body weight, height, and waist circumference were measured by physicians with standard methods and procedures. BMI was calculated as body weight divided by height squared (kg/m^2^). We also assessed history of diseases (including history of hypertension), family history of diseases, and usage of diabetes drugs (no diabetes drugs usage, using oral hypoglycemic drugs, and using insulin and/or using oral hypoglycemic drugs) in the questionnaire.

For lifestyle factors, the questions about physical activities for exercise (active exercise in order to strengthen the body) covered two aspects: frequency and duration of exercise per day. The total physical activity hours per week was calculated by multiplying the frequency and duration. Smoking questions included smoking frequency and the amount of tobacco smoked per day for smokers. For alcohol consumption, participants were asked about their drinking frequency. For dietary habit, participants were asked “In your daily diet, did you prefer meat, vegetarian diet, or balance the consumption of vegetarian diet and meat?” and “Whether you had high consumption of salt, oil, or added sugar?”.

### 2.3. Definition of Healthy Lifestyle Score

In this study, six variables were used in constructing the healthy lifestyle score (HLS): physical activity, waist circumference, BMI (kg/m^2^), dietary habits, smoking, and alcohol consumption. Each variable was given a score of 0 or 1, with 1 representing the healthy behavior category. A combined score (0–6 points) was calculated by summing the scores of these 6 factors. Healthy lifestyle factors were defined as: total house of physical activity per week >150 min; waist circumference < 90 cm for men and <85 cm for women [21]; BMI between 18.5 and 23.9 kg/m^2^ [21]; balancing the consumption of meat and a vegetarian diet, and the moderate consumption of salt, oil, and added sugar; never smoking; and no alcohol consumption. Others were defined as unhealthy. Details of the lifestyle score are described in Table 1. We also categorized the score into five groups (≤2, 3, 4, 5, and 6).

### 2.4. Definition of Glycemic Control

After an overnight fast for at least 10 h, blood samples were collected in the early morning from participants through routine physical examinations. FPG was measured with standard procedures by trained staff. We defined participants whose FPG < 7.0 mmol/L as having good glycemic control and FPG ≥ 7.0 mmol/L as having poor glycemic control, according to guidelines for the prevention and treatment of type 2 diabetes mellitus in China (2020 edition) [22].

The measurement of HbA1c was also obtained from the clinical database if patients asked for the examinations at their own expense. We defined participants whose HbA1c was <7.0% as having good glycemic control and those with HbA1c ≥ 7.0% as having poor glycemic control [22].

### 2.5. Statistical Analysis

The characteristics of the participants were expressed as means and standard deviations for the continuous variables, and as frequencies with numbers and percentages for the categorical variables. To compare the demographic and lifestyle characteristics of the participants with good glycemic control and poor glycemic control, the Wilcoxon rank-sum test was used for the continuous variables, and the chi-square test was used for the categorical variables. A logistic regression model was used to estimate the odds ratio (OR) and 95% confidence interval (CI) for the association of each lifestyle factor and the HLS with the risk of poor glycemic control. We conducted multivariable logistic regression analysis from two models. Model 1 was adjusted for age and sex. Model 2 was adjusted for age, sex, ethnic, education, marital status, duration of diabetes, hypertension, diabetes treatment, and family history of diabetes according to prior knowledge and previous studies [23,24]. Due to the sex differences in the HLSs, we also conducted analysis stratified by sex. The linear trend test used the Cochran–Armitage trend test.

To examine the robustness of the results, we also carried out a sensitivity analysis, restricting our analysis to the participants whose HbA1c values were available. Adjusted logistic regression analysis was repeated to estimate the ORs (95% CIs) for HLSs with the risk of poor glycemic control (HbA1c ≥ 7.0%).

To analyze the interaction between the healthy lifestyle factors, we constructed a 5-score HLS that was calculated using any 5 of the 6 healthy lifestyle factors. The interactive effect was examined between each 5-score HLS and the factor that was excluded from the 5-score HLS in relation to glycemic control. The test for interaction was performed by using a likelihood ratio test comparing models with and without cross-product terms.

All the analyses were performed with R software, version 4.1.1 (R Foundation). *p* values < 0.05 were considered statistically significant.

## 3. Results

### 3.1. Participant Characteristics

Of the 101,914 participants, 51.40% had glycemic levels under the target (FPG < 7.0 mmol/L). The proportions of the participants who scored 4, 5, and 6 points using the HLS were 30.79%, 26.82%, and 16.26%, respectively (Table 2).

### 3.2. Association between Risk of Poor Glycemic Control and Healthy Lifestyle Factors

As shown in Table 3, six healthy lifestyle factors, except for healthy dietary habits, were independently associated with a lower risk of poor glycemic control after adjusting for various confounding factors. Compared with the participants with the corresponding unhealthy lifestyle factors, the ORs were 0.96 (95% CI: 0.93–0.99) for healthy physical activity, 0.74 (95% CI: 0.72–0.77) for healthy waist circumference, 0.97 (95% CI: 0.94–1.00) for healthy BMI, 0.95 (95% CI: 0.89–1.01) for healthy dietary habit, 0.89 (95% CI: 0.85–0.93) for never smoking, and 0.81 (95% CI: 0.77–0.85) for never drinking alcohol, respectively.

### 3.3. Association between Risk of Poor Glycemic Control and Healthy Lifestyle Score

**Figure 2:**
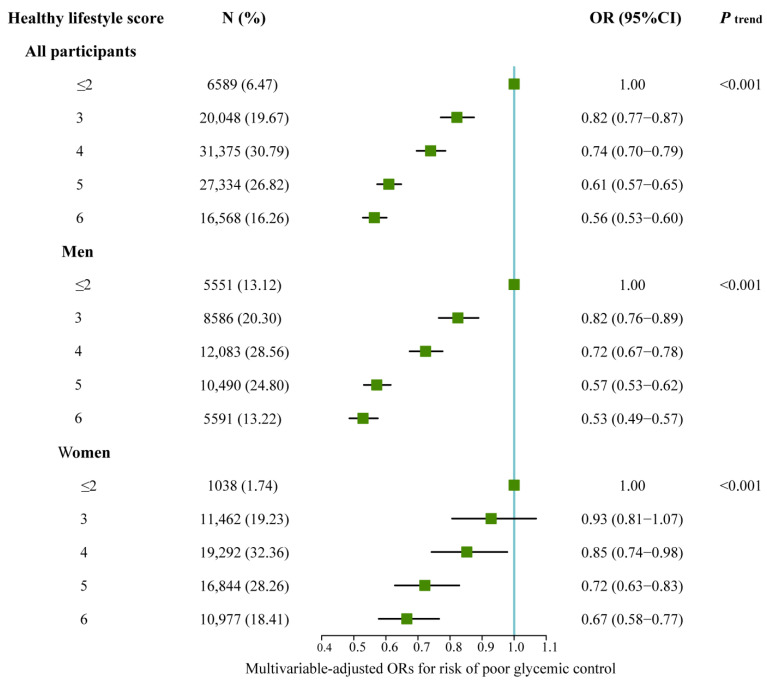
ORs (95% CIs) for risk of poor glycemic control by healthy lifestyle score in all participants, men, and women. OR, odds ratio; CI, confidence interval. Multivariable model was adjusted for age, sex, ethnic, education, marital status, duration of diabetes, hypertension, diabetes treatment, and family history of diabetes.

When considering all the healthy lifestyle factors jointly, the risk of poor glycemic control decreased significantly with an increasing number of factors (*p* for linear trend < 0.001) (Figure 2). Compared with the participants with an HLS ≤ 2, the ORs for the participants with 3, 4, 5, and 6 scores were 0.82 (95% CI: 0.77–0.87), 0.74 (95% CI: 0.70–0.79), 0.61 (95% CI: 0.57–0.65), and 0.56 (95% CI: 0.53–0.60), respectively. Sex-stratified analysis showed that the HLS was inversely associated with the risk of poor glycemic control (all *p* for linear trend <0.001). Compared with the participants with an HLS ≤ 2, the ORs for the participants with the highest score (6) were 0.53 (95% CI: 0.49–0.57) for men and 0.67 (95% CI: 0.58–0.77) for women.

### 3.4. Sensitivity Analysis

There were 4853 participants whose HbA1c was available in our study. Utilizing HbA1c as a glycemic control index, sensitivity analysis that used the same adjusted model yielded similar results. Compared with participants with an HLS ≤ 2, the ORs for the participants with 3, 4, 5, and 6 scores were 0.70 (95% CI: 0.42–1.16), 0.48 (95% CI: 0.29–0.77), 0.55 (95% CI: 0.34–0.88), and 0.47 (95% CI: 0.29–0.76), respectively. The risk of poor glycemic control decreased significantly with the increasing HLS (*p* for linear trend < 0.001).

### 3.5. Interactions between Lifestyle Factors and Glycemic Control

We found significant interactions of physical activity, waist circumference, BMI, and alcohol consumption with the 5-score HLS consisting of other lifestyle factors in relation to glycemic control (all *p* for interaction < 0.05). The ORs for the multiplicative interactions of physical activity, waist circumference, BMI, and alcohol consumption with the 5-score HLS were 1.10 (95% CI: 1.04–1.16), 0.90 (95% CI: 0.85–0.95), 0.90 (95% CI: 0.84–0.96), and 0.88 (95% CI: 0.80–0.97), respectively. Compared to those who had unhealthy lifestyle factors and a low 5-score HLS, individuals who had both healthy physical activities and a high 5-score HLS were more likely to have increased odds of good glycemic control, by 30% (95% CI: 27–33%). Similar results were seen in waist circumference, BMI, and alcohol consumption, with 33% (95% CI: 30–35%), 31% (95% CI: 29–34%) and 38% (95% CI: 34–41%), respectively. No significant interactions of dietary habit and smoking with other lifestyle factors were found (dietary habit *p* for interaction = 0.435; smoking *p* for interaction = 0.703) (Table 4).

## 4. Discussion

In this large-scale cross-sectional study, we found a strong association between combined healthy lifestyle factors and glycemic control in patients with T2DM. The healthy lifestyle included sufficient physical activity, maintaining a BMI ≤24, never smoking, no alcohol consumption, maintaining a healthy waist circumference, and eating a healthy diet. A higher HLS was associated with a lower likelihood of poor glycemic control. Significant interactions of BMI, waist circumference, alcohol consumption, and physical activities with the 5-score HLS consisting of other lifestyle factors were found in relation to glycemic control.

Our study found a significant association between combined healthy lifestyle factors and glycemic control in patients with T2DM. So far, no study has reported the relationship between combined healthy lifestyle factors and glycemic control in patients with T2DM. Only one prospective cohort study in Germany following up 866 patients with both T2DM and depression for 12 months found a nonsignificant relationship of glycemic control with total score of lifestyle behaviors including smoking, alcohol consumption, physical activities, and diet at baseline [18]. Given the influence of depression, the results could not apply to general patients with T2DM. However, some previous studies have revealed that adopting a lifestyle with a higher HLS was related to a lower risk of incidence of T2DM [25,26]. Due to the fact that T2DM is caused by impaired β cell function [6] and then leads to a high blood glucose level in patients with T2DM, this effect of combined lifestyle factors may also be responsible for glycemic control.

In the present study, we demonstrated a significant relationship between physical activities and good glycemic control, which was consistent with the results from a cross-sectional study in Korea [27]. However, some previous studies in Japan [28], Vietnam [8], and the Netherlands [7] failed to find significant associations between physical activities and blood glucose level, possibly because of insufficient sample size (183, 189, and 155 patients with T2DM, respectively). Healthy waist circumference, healthy BMI, and never having smoked were also significantly associated with good glycemic control in our study. No significant relationship was observed between dietary habits and glycemic control, which might be owing to the fact that most participants (95.45%) had healthy dietary habits. Additionally, the definition of healthy dietary habits in our study was not specific enough. For example, total energy intake was reported to be associated with glycemic control [28,29].

Previous studies tended to indicate that moderate alcohol consumption benefits glycemic control even more than no alcohol consumption [30,31]. However, our study found that participants who did not drink alcohol had better glycemic control than those who drank alcohol. The difference may result from the lack of information on the number of drinks per day and the varieties of alcohol. In most of the previous studies, the participants were Caucasian. Because of the different drinking patterns and genetic differences in alcohol-metabolizing enzymes [32], the results may not be extended to a Chinese population. It was reported that alcohol might improve insulin sensitivity and inhibit gluconeogenesis [33], which might decrease the blood glucose in low doses. However, excessive chronic alcohol consumption causes harm to health [34]. Moreover, even light alcohol drinking might increase the risk of other diseases [35]. Therefore, the recommendation of drinking alcohol for diabetics should be cautious.

There were significant interactive effects between BMI and the combination of the five other lifestyle factors for glycemic control, as well as for waist circumference, physical activities, and alcohol consumption. Our findings suggested that healthy lifestyle factors played greater roles in glycemic control among patients who had a normal BMI than among overweight patients. Similar results were also shown in waist circumference and alcohol consumption. For the interaction between physical activities and other lifestyle factors, the results indicate that patients with low levels of physical activity could benefit more from other healthy lifestyle factors. Our findings on the interactions indicate that a combined lifestyle is not just the sum of each healthy lifestyle factor alone, but also includes the dynamic interaction between them. A cross-sectional study about the associations between a combined healthy lifestyle and the incidence of a metabolic syndrome among Qatari women also found synergy between lifestyle factors [36]. Some interactions between lifestyle factors were observed in relation to the incidence of chronic diseases [15,37].

Most previous studies about HLSs included physical activities, smoking, alcohol consumption, BMI, and dietary habits [25,38,39]. In our study, we added waist circumference as one of the indicators of HLSs. Waist circumference emphasizes the importance of abdominal obesity, and it is different from BMI, which represents overall obesity. Abdominal obesity reflects visceral fat accumulation, which is related to metabolic disorders [40,41] and is related to the incidence of diabetes independent of any effect on overall obesity [42]. A possible explanation is that overall and abdominal obesity had different effects on the pathogenesis of T2DM, and overall obesity may affect insulin secretion while abdominal obesity results in worse insulin sensitivity [43].

Our study has some strengths. Few studies have examined the relationship between glycemic control and combined healthy lifestyle factors. Given that prospective and intervention studies are still lacking, our study could provide a reasonable hypothesis for the association between them. Moreover, the lifestyle factors we chose are preventable and easily measurable by the general population. Therefore, our findings offer practical suggestions to public health authorities. There were also several limitations in our study. First, due to the nature of a cross-sectional study, confounding, potential bias, and reverse causality may exist, which limits its ability to explain the causal relationship of the healthy lifestyle factors affecting the risk of poor glycemic control. Second, lifestyle behaviors were all self-reported, which could be inaccurate. Third, we defined glycemic control by FPG because of our lack of HbA1c data in most participants, but most previous studies have used HbA1c as the indicator of glycemic control [28,44]. Indeed, HbA1c reflects long-term glycemic control and is thought to be more accurate and stable, and is not impacted by stress, diet, or illness [45]. However, HbA1c may have an imperfect correlation with blood glucose [46]. Moreover, given that HbA1c is actually a weighted average of blood glucose levels for about three months and a recent glucose level contributes more [47], it may be reasonable to define glycemic control by using FPG. To examine the robustness of utilizing FPG to define glycemic control, we conducted sensitivity analysis through defining glycemic control by HbA1c. Though the sample size of participants with HbA1c was small, it presented a similar trend to FPG, suggesting the reliability of our results. Fourth, owing to the restriction of our data, some other healthy lifestyle factors that might also have associations with glycemic control were not included, such as other dietary habits including total energy intake. More prospective cohort studies and intervention studies are expected to be conducted in the future and would demonstrate the casual relationships between more combined lifestyle factors and glycemic control.

## 5. Conclusions

Our findings suggest that behavior modification may benefit glycemic control for patients with T2DM. In addition, maintaining a combined healthy lifestyle is likely to have a stronger relationship with glycemic control than maintaining healthy lifestyle factors individually. These findings underscore the importance of behavior modification and the promotion of a healthy lifestyle for effectively managing T2DM. Our study provides further scientific evidence, especially for the Chinese population, supporting the widely recognized consensus that lifestyle modification is a crucial and effective approach for the prevention and management of T2DM. Thus, public health advice should be promoted to encourage patients with T2DM to live in a healthier way including maintaining sufficient physical activity, normal waist circumference, normal BMI, and healthy dietary habits, never smoking, and not consuming alcohol. Further evidence of the longer-term impact of a healthy lifestyle on glycemic control are expected through subsequent follow-ups.

## Figures and Tables

**Table 1:** Components of lifestyle score.

Lifestyle	Category	Healthy/Unhealthy	Score
Physical activities	Total physical activity hours per week > 150 min	Healthy	1
	Total physical activity hours per week ≤ 150 min	Unhealthy	0
Waist circumference	Men: <90 cm; Women: <85 cm	Healthy	1
	Men: ≥90 cm; Women: ≥85 cm	Unhealthy	0
Body Mass Index	18.5–23.9 kg/m^2^	Healthy	1
	<18.5 kg/m^2^/≥24.0 kg/m^2^	Unhealthy	0
Dietary habits	Balanced consumption of meat and vegetarian diet, moderate salt consumption, moderate oil consumption, and moderate added sugar consumption	Healthy	1
	Prefer meat/prefer vegetarian diet/high salt consumption/high oil consumption/high added sugar consumption	Unhealthy	0
Smoking	Never	Healthy	1
	Current/former	Unhealthy	0
Alcohol consumption	Never	Healthy	1
	Current/former	Unhealthy	0

**Table 2:** Characteristics of participants.

	Overall (*n* = 101,914)	Glycemic Control ^a^		*p* Value ^b^
		Good (*n* = 52,381)	Poor (*n* = 49,533)	
Sex, *n* (%)				0.002
Men	42,301 (41.51)	21,494 (41.03)	20,807 (42.01)	
Women	59,613 (58.49)	30,887 (58.97)	28,726 (57.99)	
Age, years, mean ± SD	65.86 ± 10.19	66.13 ± 10.38	65.58 ± 9.99	<0.001
Duration of diabetes, years, mean ± SD	5.93 ± 5.64	5.57 ± 5.40	6.30 ± 5.86	<0.001
Ethnicity (Han), *n* (%)	101,744 (99.83)	52,297 (99.84)	49,447 (99.83)	0.659
Education, *n* (%)				<0.001
Illiteracy	27,866 (27.34)	14,081 (26.88)	13,785 (27.83)	
Primary and high school education	41,439 (40.66)	21,493 (41.03)	19,946 (40.27)	
College education and above	32,064 (31.46)	16,483 (31.47)	15,581 (31.46)	
Marriage (Married), *n* (%)	90,505 (88.81)	46,448 (88.67)	44,057 (88.94)	0.241
Family history of diabetes, *n* (%)	7141 (7.01)	3407 (6.50)	3734 (7.54)	<0.001
Hypertension, *n* (%)	57,804 (56.72)	30,791 (58.78)	27,013 (54.54)	<0.001
Waist circumference, cm, mean ± SD	85.98 ± 25.83	85.36 ± 34.48	86.64 ± 10.71	<0.001
Body mass index, kg/m^2^, mean ± SD	24.68 ± 3.98	24.52 ± 3.71	24.84 ± 4.24	<0.001
Sufficient physical activity	59,003 (57.89)	30,470 (58.17)	28,533 (57.60)	0.068
Smoking, *n* (%)				<0.001
Never	84,568 (82.98)	44,164 (84.31)	40,404 (81.57)	
Former	4142 (4.06)	2003 (3.82)	2139 (4.32)	
Current	13,204 (12.94)	6214 (11.87)	6990 (14.11)	
Alcohol consumption, *n* (%)				<0.001
Never	91,368 (89.65)	47,636 (90.94)	43,732 (88.29)	
Former	1686 (1.65)	807 (1.54)	879 (1.77)	
Current	8860 (8.69)	3938 (7.52)	4922 (9.94)	
Dietary habits, *n* (%)				0.022
Balanced meat and vegetarian diet	98,431 (96.58)	50,618 (96.63)	47,813 (96.53)	
Prefer meat	973 (0.95)	458 (0.87)	515 (1.04)	
Prefer vegetarian diet	2510 (2.46)	1305 (2.49)	1205 (2.43)	
High salt consumption, *n* (%)	1084 (1.06)	537 (1.03)	547 (1.10)	0.230
High oil consumption, *n* (%)	392 (0.38)	184 (0.35)	208 (0.42)	0.086
High sugar consumption, *n* (%)	240 (0.24)	125 (0.24)	115 (0.23)	0.882
Diabetes treatment				0.018
None	26,922 (26.42)	13,868 (26.48)	13,054 (26.35)	
Oral hypoglycemic drugs	69,074 (67.78)	35,577 (67.92)	33,497 (67.63)	
Insulin or insulin + oral hypoglycemic drugs	5918 (5.81)	2936 (5.61)	2982 (6.02)	
Fasting blood glucose, mmol/L, mean ± SD	7.86 ± 5.05	5.87 ± 0.77	9.96 ± 6.58	<0.001

SD, standard deviation. ^a^ Good glycemic control: FPG < 7.0 mmol/L; Poor glycemic control: FPG ≥ 7.0 mmol/L. ^b^ The Wilcoxon rank-sum test was used for the comparison of continuous variables, and the chi-square test was used for the comparison of categorical variables.

**Table 3:** Risk of poor glycemic control by healthy lifestyle factors (*N* = 101,914).

	Cases, *n* (%)	OR (95% CI) ^a^	
		Model 1	Model 2
Physical activity ^b^			
Unhealthy	42,911 (42.11)	1.00	1.00
Healthy	59,003 (57.89)	0.98 (0.96–1.01)	0.96 (0.93–0.99)
Waist circumference ^c^			
Unhealthy	44,523 (43.69)	1.00	1.00
Healthy	57,391 (56.31)	0.77 (0.74–0.79)	0.74 (0.72–0.77)
BMI ^d^			
Unhealthy	58,146 (57.05)	1.00	1.00
Healthy	43,768 (42.95)	0.99 (0.96–1.02)	0.97 (0.94–1.00)
Dietary habit ^e^			
Unhealthy	4636 (4.55)	1.00	1.00
Healthy	97,278 (95.45)	0.96 (0.90–1.02)	0.95 (0.89–1.01)
Smoking ^f^			
Unhealthy	17,346 (17.02)	1.00	1.00
Healthy	84,568 (82.98)	0.88 (0.84–0.91)	0.89 (0.85–0.93)
Alcohol consumption ^g^			
Unhealthy	10,546 (10.35)	1.00	1.00
Healthy	91,368 (89.65)	0.81 (0.78–0.85)	0.81 (0.77–0.85)

OR, odds ratio; CI, confidence interval; BMI, body mass index. ^a^ Model 1 was adjusted for age and sex; Model 2 was further adjusted for ethnicity, education, marital status, duration of diabetes, hypertension, diabetes treatment, and family history of diabetes. ^b^ Healthy: total physical activity hours per week > 150 min; Unhealthy: total physical activity hours per week ≤ 150 min. ^c^ Healthy: waist circumference < 90 cm for men and <85 cm for women; Unhealthy: waist circumference ≥ 90 cm for men and ≥85 cm for women. ^d^ Healthy: BMI between 18.5 and 23.9 kg/m^2^; Unhealthy: BMI below 18.5 or over 24.0. ^e^ Healthy: Balanced consumption of meat and vegetarian diet and moderate consumption of salt, oil, and added sugar; Unhealthy: prefer meat/prefer vegetarian diet/high salt consumption/high oil consumption/high added sugar consumption. ^f^ Healthy: never smoking; Unhealthy: former or current smoking. ^g^ Healthy: never drinking alcohol; Unhealthy: former or current alcohol drinking.

**Table 4:** Interaction of one healthy lifestyle factor and other factors in relation to risk of poor glycemic control.

	5-Score HLS ^a^		*P_interaction_*	ORs (95% CIs) for Interaction
	Low (0–2)	High (3–5)		
Physical activity ^b^			0.001	1.10 (1.04–1.16)
Unhealthy	1.00	0.70 (0.67–0.73)		
Healthy	0.92 (0.88–0.95)	0.70 (0.67–0.73)		
Waist circumference ^c^			<0.001	0.90 (0.85–0.95)
Unhealthy	1.00	0.94 (0.91–0.99)		
Healthy	0.79 (0.75–0.83)	0.67 (0.65–0.70)		
BMI ^d^			0.001	0.90 (0.84–0.96)
Unhealthy	1.00	0.80 (0.77–0.83)		
Healthy	0.95 (0.90–1.01)	0.69 (0.66–0.71)		
Dietary habit ^e^			0.435	0.95 (0.83–1.08)
Unhealthy	1.00	0.79 (0.69–0.90)		
Healthy	0.97 (0.89–1.06)	0.72 (0.66–0.79)		
Smoking ^f^			0.703	0.99 (0.92–1.06)
Unhealthy	1.00	0.78 (0.72–0.83)		
Healthy	0.85 (0.81–0.90)	0.65 (0.62–0.68)		
Alcohol consumption ^g^			0.011	0.88 (0.80–0.97)
Unhealthy	1.00	0.87 (0.79–0.96)		
Healthy	0.81 (0.77–0.86)	0.62 (0.59–0.66)		

OR, odds ratio; CI, confidence interval; HLS, healthy lifestyle score; BMI, body mass index. ^a^ Data are presented as OR (95% CI). Model was adjusted for age, sex, ethnic, education, marital status, duration of diabetes, hypertension, diabetes treatment, and family history of diabetes. ^b^ Healthy: total physical activity hours per week > 150 min; Unhealthy: total physical activity hours per week ≤ 150 min. ^c^ Healthy: waist circumference < 90 cm for men and <85 cm for women; Unhealthy: waist circumference ≥ 90 cm for men and ≥85 cm for women. ^d^ Healthy: BMI between 18.5 and 23.9 kg/m^2^; Unhealthy: BMI below 18.5 or over 24.0. ^e^ Healthy: Balanced consumption of meat and vegetarian diet and moderate consumption of salt, oil, and added sugar; Unhealthy: prefer meat/prefer vegetarian diet/high salt consumption/high oil consumption/high added sugar consumption. ^f^ Healthy: never smoking; Unhealthy: former or current smoking. ^g^ Healthy: never drinking alcohol; Unhealthy: former or current alcohol drinking.

## Data Availability

The data presented in this study are available on request from the corresponding author.
